# Patient perspectives on data sharing regarding implementing and using artificial intelligence in general practice – a qualitative study

**DOI:** 10.1186/s12913-023-09324-8

**Published:** 2023-04-04

**Authors:** Josefine Graabaek Mikkelsen, Natasha Lee Sørensen, Camilla Hoffmann Merrild, Martin Bach Jensen, Janus Laust Thomsen

**Affiliations:** Center for general practice, Aalborg University, Aalborg, Denmark

**Keywords:** Patient perspectives, Trust, Patient-GP relationship, Artificial intelligence, Data sharing, General practice

## Abstract

**Background:**

Due to more elderly and patients with complex illnesses, there is an increasing pressure on the healthcare system. General practice especially feels this pressure as being the first point of contact for the patients. Developments in digitalization have undergone fast progress and data-driven artificial intelligence (AI) has shown great potential for use in general practice. To develop AI as a support tool for general practitioners (GPs), access to patients’ health data is needed, but patients have concerns regarding data sharing. Furthermore, studies show that trust is important regarding the patient-GP relationship, data sharing, and AI. The aim of this paper is to uncover patient perspectives on trust regarding the patient-GP relationship, data sharing and AI in general practice.

**Method:**

This study investigated 10 patients’ perspectives through qualitative interviews and written vignettes were chosen to elicit the patients (interviewees) perspectives on topics that they were not familiar with prior to the interviews. The study specifically investigated perspectives on *1) The patient-GP relationship, 2) data sharing regarding developing AI for general practice*, and *3) implementation and use of AI in general practice* using thematic analysis. The study took place in the North Denmark Region and the interviewees included had to be registered in general practice and be above 18 years in age. We included four men between 25 to 74 years in age and six women between 27 to 46 years in age.

**Results:**

The interviewees expressed a high level of trust towards their GP and were willing to share their health data with their GP. The interviewees believed that AI could be a great help to GPs if used as a support tool in general practice. However, it was important for the interviewees that the GP would still be the primary decision maker.

**Conclusion:**

Patients may be willing to share health data to help implement and use AI in general practice. If AI is implemented in a way that preserves the patient-GP relationship and used as a support tool for the GP, our results indicate that patients may be positive towards the use of AI in general practice.

**Supplementary Information:**

The online version contains supplementary material available at 10.1186/s12913-023-09324-8.

## Background

Many healthcare systems are currently under pressure because of an increasing longevity of life leading to more elderly and a growing number of patients with complex multimorbidity [1]. General practitioners (GPs) and other healthcare professionals in general practice must work faster to keep up with the increasing workload. At the same time, the requirements for documentation and administration are increasing and general practice in particular feels the pressure since the patients’ first point of contact with the healthcare system happens here, and because general practice works as gatekeeper to the secondary sector. The increasing pressure on general practice is resulting in less time for talking to and examining the patients [2, 3].

In recent years, the development and use of data-driven artificial intelligence (AI) has also increased dramatically. In 2019 the Danish government proposed a national strategy for implementing AI in the healthcare system. The strategy was proposed because of the possibility for decision support tools and streamlining the healthcare system through their use and the sharing of the large amount of data which is available in the healthcare system [3]. However, existing literature shows that when it comes to sharing data, patients are concerned with aspects regarding confidentiality, security, privacy, and trust in the unit or organization conducting the research [4–6]. Patients seem most willing to share data with the established healthcare system and least likely to share data with insurance companies, companies associated with biotechnology, and pharmaceutical products [4]. With AI added to the equation, a new perspective appears. Some studies have shown that patients are more receptive to the use of AI if it is implemented and used in a way that preserves the patient-doctor relationship, partly due to fear of replacement of humans and partly because AI challenges the humanistic aspect of health care [7, 8]. It has been suggested that the possibility for continuity and longevity of contact with the GP, along with the broad range of health-related concerns that people seek care for in general practice makes the patient-GP relationship particularly important in general practice [9]. It has been argued that to provide the patient with good care and effective encounters, there is a need for a healthy patient-GP relationship based on trust and good communication [10, 11]. Trust is needed to create an atmosphere in which honest and good communication can arise, and it has been suggested that the quality of the patient-GP relationship can be directly related to the trust between them [12].

In relation to medical doctors there is an expected level of competence since doctors’ medical degree and their right to practice mark a passage to being a professional [13], and due to their level of education and competences doctors are awarded status, power and authority in society [14]. In fact, a study has shown that doctors are one of the most trusted professions and have the highest status in society both among the population and other professions [14]. However, beginning around the turn of the 21st century and continuing to present day, there have been changes in the way of thinking about trust in relation to doctors as a profession, including GPs themselves. Studies point towards reduced professional status as a driving force in and around medicine [15, 16]. This may partly be because of the introduction of new medical technologies that can limit doctors’ fields of expertise and make them more dependent on technology [16]. In addition to this, online health information is now freely available, and the use of Google searching for health advice is widely used [17]. This access to information has made it possible for people to educate themselves online about medical conditions or health-related issues without having to consult with their GP [18].

Another important aspect regarding trust in the patient-GP relationship is the sharing of health data. Trust regarding sharing health data is important in order to implement and use AI in general practice in a way with which patients’ feel comfortable and currently, sharing and reuse of patient’s health data has shown to be a sensitive matter that has developed into public issues in some European countries [19]. Furthermore, trust in AI is a complex matter. In an article on a theory of trust for AI in healthcare, it is pointed out that the achievement of trust in AI in a healthcare setting can be even more complex than trust in AI in general, which can partly be explained by the limited existence of public literature about AI in a general practice context [20]. It is argued that trust in technologies should be complemented by trust in those producing the technology and that such trust can only be achieved if the companies producing the technology are transparent about their data use and policies. When data is used to promote AI, the people providing the data should be aware of how their data are handled, stored, and how it is being used [21]. Although trust in AI is a complex matter, some patients view the use of innovative techniques such as AI for processing health data as an opportunity for GPs to benefit from the use of AI solutions in the medical process [22]. Furthermore, research points out that some patients perceive AI as a diagnostic tool that can increase the diagnostic speed, which they view as beneficial and possibly lifesaving, an understanding that is based on AI’s ability to draw on more data or experience than humans [8].

The above observations call for more research on patient perspectives on data sharing specifically regarding AI in general practice, in order to expand our understanding of the potential challenges surrounding this. Therefore, the aim of this paper is to uncover new patient perspectives on the importance of trust regarding the patient-GP relationship, data sharing and AI in general practice.

## Methods

The authors have followed the Standards for Reporting Qualitative Research (SRQR) table guidelines for reporting qualitative studies when conducting the methods section [23].

### Researchers’ characteristics and reflexivity

The first author is a Ph.D. student with a background in health promotion and psychology, and patient perspectives is therefore a fundamental focus point when investigating implementing and using technology in general practice. The second author is a Ph.D. student with a background in medicine with industrial specialization and is preoccupied with AIs place in the health care system, especially regarding decision support. The third author is a researcher with specialty in qualitative methods and is also focused on patient perspectives regarding implementing technology in general practice. The fourth author is a professor and GP and is preoccupied with using technology as a support tool for general practice. The final author is a professor and GP and is focused on the implementation of technology in general practice. All the authors have the assumption that technology can be helpful in general practice, if it is implemented and used in an ethical manner that comply with the law regarding data protection.

### Context

The study took place in the North Denmark Region, and in Denmark the healthcare system is public and free for all citizens financed by taxes and almost all patients are listed with a GP. The GPs are paid by capitation and fee-for-service reimbursement [24]. GPs are responsible for providing patients with palliative care and can refer patients to specialist treatment if needed. GPs are also responsible for patient care 24hours a day [25].

### Sampling strategy

The strategy was to get a broad perspective with both male and female patients in different age groups and professions. Other than that, the inclusion criteria for patients were that they must be Danish citizens above 18 years of age and registered at a general practice clinic.

We only included a sample of 10 interviewees in the study, since we included based on the concept information power, which relates to the study purpose, quality of dialogue and analysis strategy [26], and when the same perspectives kept coming up we felt a data saturation.

### Data collection methods and interviewees

The study consists of 10 patient interviews carried out between October 2019 and January 2022. Nine interviews were carried out by the second author and one interview by the first author. All the patients (interviewees) were recruited from the North Denmark Region through different Facebook groups. A Facebook post explaining the project was made and movie tickets were offered as compensation. The post was published on different city groups in North Denmark Region as well as the “*AAU (Aalborg University) seek, find and become test subject*” Facebook group.

Each interview lasted 30 to 45 minutes and the interviewees decided where the interviews were held. The interviewees ended up consisting of four men 25 to 74 years in age, and six women 27 to 46 years of age at the time of the interview. The ten interviewees’ educational background years ranged from short to medium to long and some were students. See Table 1 regarding interviewee information.

**Table 1:**
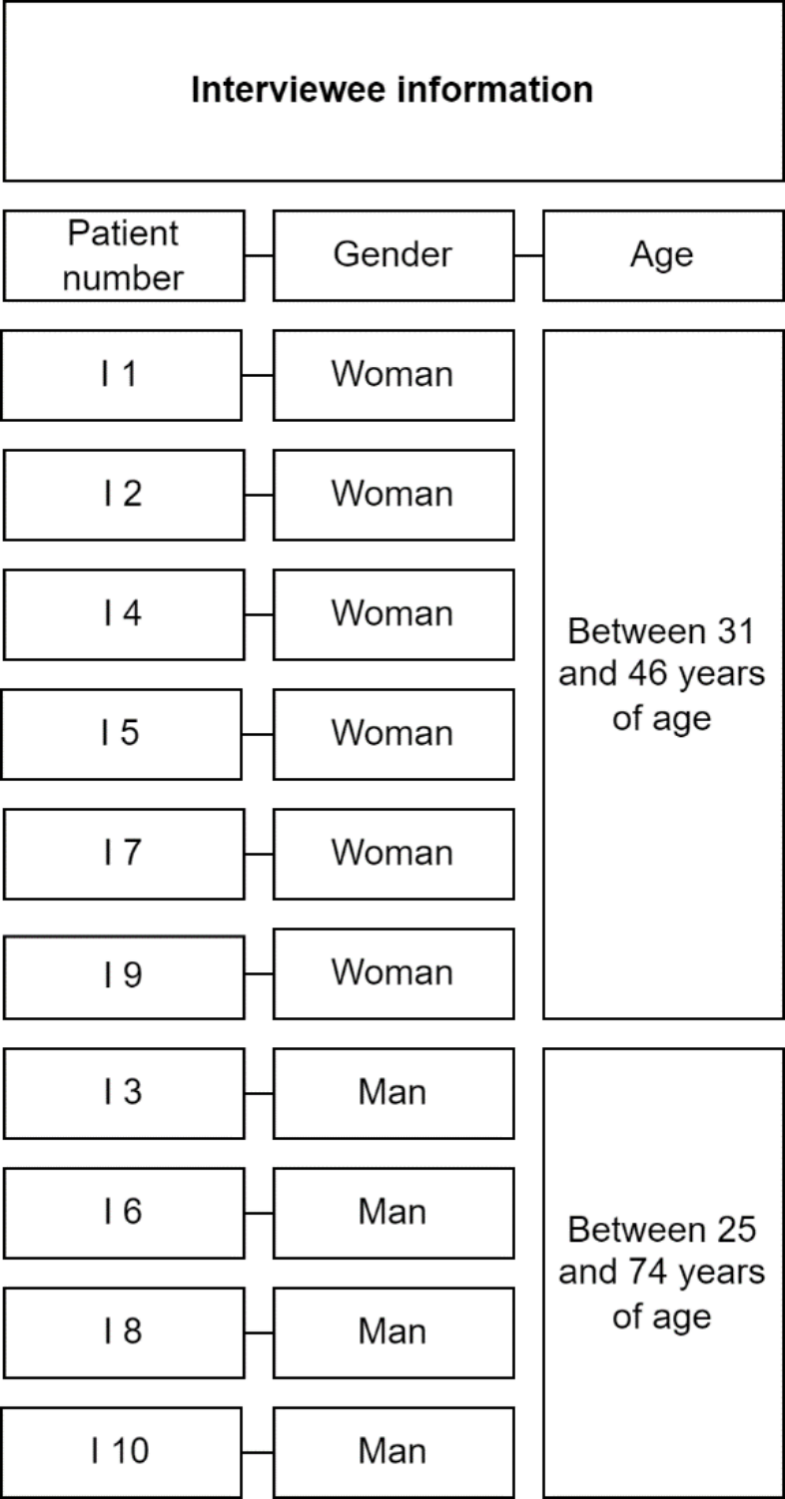
The patient number shown in the table will be used for reference in the analysis

### Data collection instruments and technologies

We used an audio recorder for data collection, and all interviews were transcribed verbatim by the first author. The Nvivo13 software program was used to transcribe and code the data by marking and naming selections of text within all datasets.

### Vignette method

The interviews were conducted with the vignette method, which is a methodological tool that entails a written, visual, or crafted incident and then presenting it to the interviewees in order to elicit their opinions and perspectives [27]. Vignettes are designed to simulate events in a hypothetical way to investigate how people might react to such events [28]. Vignettes seek the understanding of people’s attitudes, perceptions, and beliefs, especially concerning sensitive subjects such as healthcare [29]. For this study, three vignettes were developed in written form and the patient referred to in the vignettes is fictional (Appendix 1). We found this method particularly relevant for this study since patients in Denmark do not have much experience with AI in general and do not know much about data sharing in relation to implementing and using AI in general practice. Even though we refer to data-driven AI, we were not explicit about this towards the interviewees, who were only presented with the term “artificial intelligence”, since they probably would not have gained any further knowledge from the information, since they did not know much about AI in general.

Right before the interviews started, the interviewees were asked about their age, where they live, education, and profession. We used a semi-structured interview guide (Script, Appendix 2) to enable unforeseen perspectives and creativity to come forward. The script consisted of different themes with multiple questions for each theme. The interviews started with a vignette and from there on the questions began. The first line of questions regarded understanding of the first vignette, health data, and AI. After the second vignette, the questions concerned understanding of the vignette, when should data be shared/made available, sensitivity of data, and data security. After the third and final vignette, the questions involved understanding of the vignette and trust in the use of AI in general practice.

### Data processing and data analysis

**Fig. 1:**
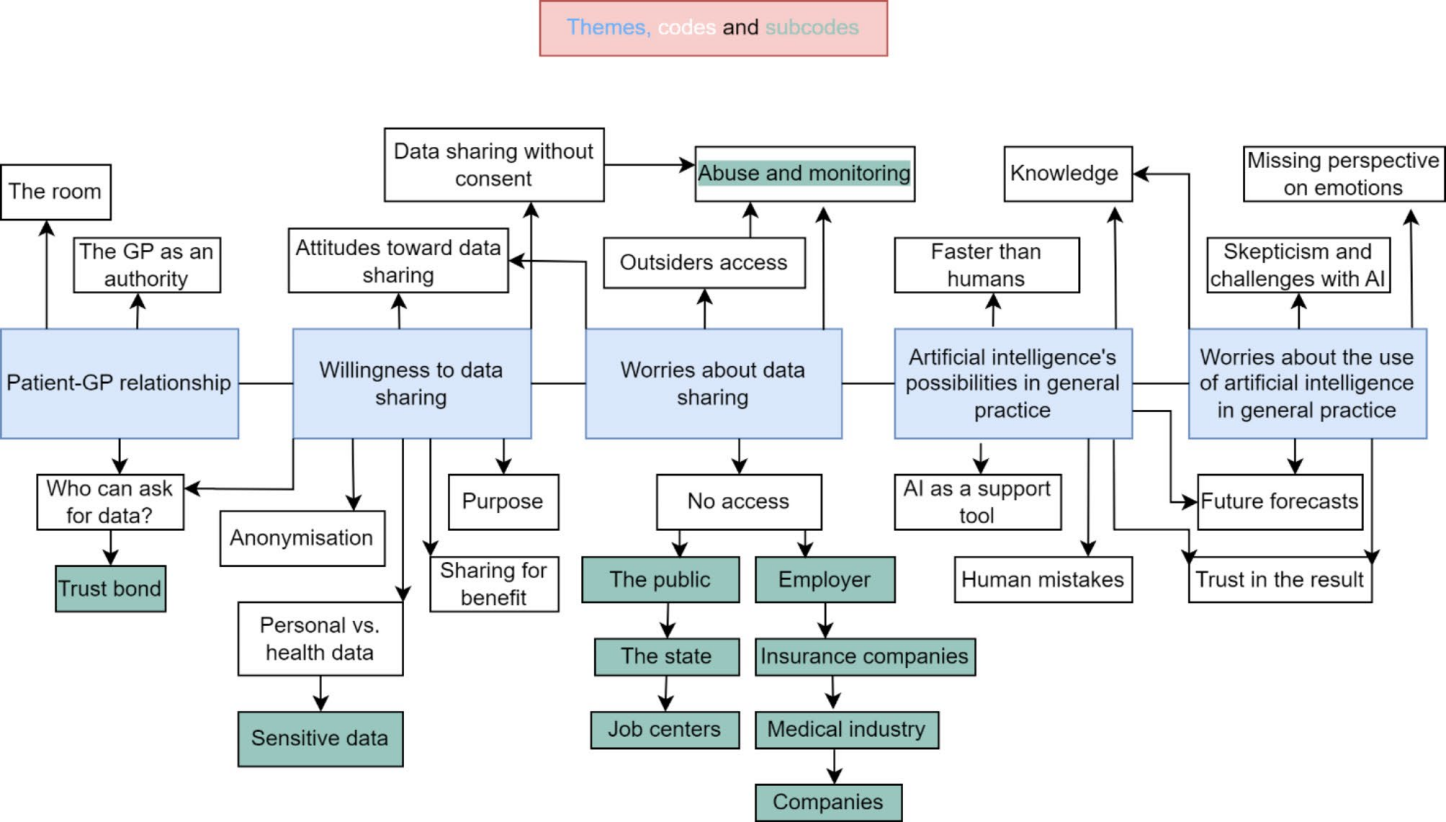
The figure illustrates the five themes and their relations to the codes and subcodes. The codes with a direct arrow from one or more of the themes are main codes while the codes with arrows from other codes are subcodes of these

An inductive approach was applied to analyze the data and we used member checking as a technique to enhance trustworthiness and credibility. The first author carried out a thematic analysis using the six phases of analysis [30], with inputs and feedback from the rest of the authors. Transcribing the data is the first key phase in a thematic analysis when interpreting data, and while listening to the interviews and reading, ideas and notes were made. The second phase regarded generating the initial codes by using an open coding approach. In this case, the coding began by re-reading all the transcriptions and review notes, and then looking for patterns and perspectives that occurred multiple times and in several interviews. Then the third phase began, which involved searching for themes by analyzing the codes, and sorting them into themes, see Fig. 1 below. In practice this meant exploring the codes’ relations to each other and seeing how they fit into the overall story about the data. The fourth phase was reviewing the themes, the dataset was re-read, and then the fifth phase started, which involved defining and naming the themes, the final themes were defined and named based on the codes and subcodes found in Fig. 1. The sixth and last phase regarded producing the report.

The themes ended up being 1) Patient-GP relationship, 2) Willingness for data sharing, 3) Worries about data sharing, 4) Artificial intelligence’s possibilities in general practice, and 5) Worries about the use of artificial intelligence in general practice.

## Results

### Patient-GP relationship

One of the main findings of the study was that the interviewees felt comfortable around their GP and had general trust in them. This was reflected in the interviewees’ willingness to share their data if their GP asked them to. The interviewees’ willingness to share in this context indicates that they trust their GP, and five of the interviewees, two men and three women, mention that they have a close relationship with their GP or believe there is some kind of trust bond between patients and their GP. When asked about their feelings towards sharing data with the GP, four of the interviewees, two men and two women, began wondering about the GPs authority and one of the interviewees stated: “*You have trust towards your GP, and they are kind of an authority person, they know best, and you have to trust that. It is also a form of power*” (I 9). The interviewees that thought of their GP as an authority felt that authority contributed to them feeling in safe hands regarding data sharing. However, one interviewee expressed concerns about feeling pressured to please the GP and the fact that patients are deeply dependent on their GP: “*In that situation one will feel pressured to please the GP and say, “oh yes of course*”” (I 6). A specific concern form one of the interviewees related to the reduction in people’s trust in authorities in general: “*There has probably been a movement in relation to how much people believe in authorities. I can feel that in my profession (as a policeman), so the level of how much a person believe in authority will probably decrease over the years I’m afraid*” (I 10).

### Willingness for data sharing

The purpose behind data sharing was relevant for the interviewees, and they all found implementing and using AI in general practice to be a purpose for which they would be willing to share their data. One interviewee noted: “*Both developing AI to help the GP and cure illnesses are in the same category. They are both equally good causes that I would share my data for*” (I 3). Another perspective appeared when the interviewees were asked about the difference between sharing health data and personal data such as e-mail address, home address and social security number. Three of the interviewees, two men and one woman, felt they had nothing interesting in their health journal and nothing to hide, so they had no problem sharing their health data. However, they did not like the thought of sharing their personal data, since that was more sensitive to them, as one of the interviewees stated: “*My medical record is really boring, so my personal information would probably be more critical to share, although I share them the most already*” (I 3). When the interviewees were asked about what kind of health data are more sensitive than other data, early retirement and mental illnesses came up several times. Two of the interviewees, a man and a woman, referred to mental illnesses such as depression as taboo. One of the interviewees noted: “*I could imagine that the depression would be a sensitive subject for her [the woman in the vignette] because it is a mental illness, there is probably also many emotions involved*” (I 1). When asked about data sharing in relation to sensitive data, eight of the interviewees, three men and five women, expressed that having sensitive health information in their journal would make them more skeptical and influence their decision to share data in a negative way.

However, a common opinion among six of the interviewees, three men and three women, was that the assurance of anonymization made it feel safer and easier for them to agree to sharing their data. However, two of the interviewees, two women, still had reservations towards the use of their data concerning access to the data and how the data key could be recreated after anonymization or pseudonymization. Therefore, the interviewees wanted to be more involved in the actual process of data sharing. The interviewees were also asked about the topic of data sharing without consent, and many different opinions arose. An opinion between three of the female interviewees was that data should not be shared without consent no matter what, but four of the interviewees, one man and three women, felt that there could be exceptions if something terrible was to happen, as one interviewee noted: “*It should only be in extreme cases… an epidemic that could hurt half of the Danish population*” (I 3).

Another reservation that four of the interviewees, two men and two women, expressed regarded fear of sharing their data due to the possibility for monitoring, deprivation of liberty, general misuse, hacking or further data sharing than they had agreed to. The interviewees that were willing to share their data still expressed some reservations regarding security and who would be able to access their data. One interviewee noted: “*There has to be some sort of limit, so everyone cannot snoop around*” (I 4). Six of the interviewees, two men and four women, had concerns specifically regarding commercial or private companies, employers, the public, job centers, the state, and the medical industry. The concerns seemed to come from the interviewees fear of the above-mentioned misuse of their data. Four of the interviewees, two men and two women, pictured themselves as examples and got worried about what would happen to them if their data were shared. One interviewee expressed: “*I have a sleep-app, that measures when I go to bed and when I wake up, and sometimes you can go to bed late and I have a sleeping illness, and I think what if the job center discovered that… What can they use it for? It would probably not benefit me*” (I 7).

### Artificial intelligence’s possibilities for general practice

All of the interviewees could see possibilities for using AI in general practice and the most commonly perceived benefit among all the interviewees was if the GP could use AI as a support tool. Half of the interviewees expressed that they would even feel safer if the GP used AI as a support tool when diagnosing. This opinion was partly explained by the fact that GPs are quite busy and thus the risk of the GPs overlooking something important regarding patients’ health was perceived as a possibility that AI could prevent. One interviewee stated: “*Maybe AI could help keeping track of patient records… AI could get an overview fast and see that this patient has now shown these symptoms for the fifth time, so maybe it is time to look into that instead of the GP missing it*” (I 2). Three female interviewees mentioned that humans, including GPs, make mistakes, which they viewed as another good reason to use AI as a support tool. For example, one interviewee stated: “*We are only humans, so is the GP. Making an extra check with AI would make me feel safer*” (I 5). However, the interviewees still felt that the GP should be the primary decision maker and AI should only be used as a support tool in general practice. Some of the positive perspectives among five of the interviewees, one man and four women, regarding AI in general practice revolved around AI’s ability to find patterns in data, develop overviews, work faster than the GP, and improve the quality of problem solving. However, when asked if and how AI would affect the GPs’ workflow, one interviewee noted: “*It is a radical change compared to how health is being practiced now*” (I 3). Four other interviewees, two men and two women, thought that with time patients would begin to trust AI, especially if AI proved to be continuously correct and AI received more exposure in society. A couple of the interviewees compared it to the fact that nowadays people are used to other technologies such as phones and tablets, and with time it could be the same with AI.

A topic that generated many different perspectives was if AI could be used to detect future illnesses. The different perspectives could be explained by the fact that the interviewees had never thought about this scenario before. If AI detected an illness three of the interviewees, two men and a woman, would want to know about it instantly, while two other female interviewees did not. Those who wanted to know right away, wanted to know so they could change their ways and hopefully prevent the illness from progressing. One interviewee noted:” It *would be amazing if the illness could be detected by AI and prevented earlier, instead of trying all sorts of things before knowing what it actually is*” (I 8). Two female interviewees perceived the possibility of AI detecting future illnesses as an ethical dilemma. The ones who did not want to know thought knowing could lead to anxiety, paranoia, not being able to enjoy the present moment, or thought it would get too expensive for the healthcare system due to over-diagnosis and increased focus on disease prevention.

### Worries about the use of AI in general practice

A worry regarding AI among six of the interviewees, three men and three women, concerned AI taking over the GPs’ position and the patients losing their relationship with the GP. As one interviewee stated: “*A person does not have a trustful relationship with a machine*” (I 3). Related worries among the interviewees dealt with the fear that using AI in general practice could change the GPs’ role from health care provider to data collector or give AI too much influence and possibly outsmart the GPs. When talking about what AI could possibly be used for in general practice in the future, three of the interviewees, one man and two women, expressed concern about AI not being able to detect emotional states and show a rather one-sided perspective only based on non-emotional health data. Because of this worry, the interviewees did not think AI could ever provide a complete overview of the patients, and therefore they felt like the GP should be critical towards using it. Lack of trust in AI was also reflected in one of the interviewees’ statements: “*AI is not a living creature and depending on what you feed it with, it can learn different things, so it is important to be critical*” (I 2). The critical mindset towards AI was also expressed when the interviewees were asked about their trust in a potential diagnosis set by AI. Two female interviewees were directly against the possibility that AI could one day make diagnose by itself, while two others, a man and a woman, were concerned about trusting AI’s “opinion” and whether the AI dataset could be large enough to provide a precise diagnosis.

## Discussion

This study’s aim was to uncover patient perspectives on trust regarding the patient-GP relationship, data sharing and AI in general practice and the results uncovers many different perspectives viewpoints. The study provides some insights into how AI and data sharing feed into the patient-GP relationship, which the interviewees viewed as important. The interviewees generally had a high level of trust towards their GP, but one of the interviewees expressed concern regarding a general reduction in people’s trust in authorities, which correlates with previously mentioned study findings, that points toward a reduced professional status among doctors [17, 18]. Opposite to this perspective, the ten interviewees felt comfortable enough to share their health data with the GP for a research project regarding using AI in general practice. However, the interviewees had concerns especially in relation to outsiders’ access to their health data and lack of privacy and AI not being able to detect emotional states. Furthermore, the interviewees were afraid that using AI in general practice would change the patient-GP relationship, and they did not trust AI on its own, although they believed AI would be a beneficial support tool for GPs and general practice.

The patient-GP relationship was viewed as very important for the interviewees and they expressed fear that AI could influence the trust between them and the GP stating that people cannot trust a machine and that people must be critical towards AI. As mentioned previously the patient-GP relationship is emphasized as important in several other studies [9–12]. However, an Australian study considers that AI could be perceived as more ethical than a human GP, since it can be more effective, unbiased, and not prone to human fallibility. Furthermore, AI will not get tired after a long shift like a human GP will [31]. Some of the mentioned perspectives were also considered by the interviewees, but they still trusted the GP much more than AI regarding general practice care. However, some users may be happy to take an algorithms claims based on trust, but it has been suggested that a trustworthy algorithm should be able to show how it is working to those who want to understand how it came to its conclusions and interested parties should be able to assess the reliability of such claims [32].

The interviewees in this study expressed different concerns regarding data sharing, and a study consisting of focus groups with patients from 16 different countries showed similar concerns about data sharing, especially regarding insurance companies and employers gaining access to the data [33]. Other studies from the United Kingdom revealed concerns associated with lack of privacy and fear of private companies using the data for profit [34, 35]. Insurance and private companies are not the only ones that patients are concerned with regarding data sharing. A study investigating the social, ethical, and legal issues that impact on genetic information and testing in employment in Europe, shows that there has also been anxiety among the public regarding employers using personal genetic data to discriminate employees who are at risk of a certain disease or condition [36]. This all suggests a general fear regarding the misuse of data on multiple levels, which could be problematic if AI should be implemented and used in general practice, since the possibility for effective decision support through AI is linked to sharing large amounts of health data [37].

In this study, the interviewees considered AI to be a beneficial support tool that could increase the diagnostic speed. Half of the interviewees even expressed that they would feel safer if the GP used AI as a support tool when diagnosing. The interviewees’ positive attitudes towards using AI as a support tool correlates with the increasing use of new medical technologies that, as previously argued, can possibly make GPs more dependent on technology and the prospect of GPs being more dependent on technologies could lead to reduced professional status [18]. However, the interviewees still wanted the GPs to be the primary decision makers. Studies investigating America’s, the United Kingdom’s and the Dutch’s population views on AI in healthcare settings show similar results. Here, the patients believed that AI should be used as a support tool rather than a primary decision maker, which suggests that people are not interested in pursuing decision making pathways without a human involved [9, 31, 38]. The consensus that AI should not be the primary decision maker suggests that medical technologies are not perceived as being able to take the GPs’ place. Some of the interviewees in this study were sure that with time they would trust AI, especially if AI proved to be correct time and time again and received more exposure in society. The interviewees reasoned that people would get used to AI as they did with iPhones and tablets over a decade ago. This opens the possibility that if AI is implemented in general practice in a way that preserves the patient-GP relationship and is used primarily as a support tool for the GP, patients may trust AI eventually.

### Strengths and limitations

This study investigates patient perspectives on data sharing regarding implementing and using AI in general practice, which opens an important perspective that has received little attention until now. Therefore, this study could serve as a steppingstone for the creation of guidelines on how to implement and use AI in general practice in a patient-friendly way.

The interviewees did not have prior experience with or much knowledge on data sharing regarding the use of AI in general practice or AI in general, and therefore the vignette method was considered a good dialogue starter. However, the interviewees’ limited knowledge on the topics meant that many of the interviewees changed their mind or expressed different opinions on the same topic during the interviews. This may partly be explained by the fact that the interviewees were introduced to different examples and scenarios in the vignettes they read during the interviews. Using vignettes in this way can lead to the interviewees getting information overload [28]. To prevent information overload, the vignettes only used short sentences.

Methodical we found three aspects to discuss. The first one being that the study was conducted in Denmark among Danish patients and therefore the results are probably most relevant and transferable to countries with similar healthcare systems. However, countries with similar healthcare systems can be different regarding access and availability when it comes to patient’s possibilities for making appointments with a GP. The GPs’ role and status as primary physician and therefore a stable and relatable figure is widespread, and GPs are universally highly educated. However, in countries that face problems regarding access and availability to GPs, it is much harder for the patients to develop trust towards their GP, since the continuity and longevity of contact with the GP is a big part of creating the patient-GP relationship and the trust between them [9–11].

Second, a greater number of patient perspectives may have identified additional insights. However, the 10 interviews we carried out were in depth, and offered the patients a chance to truly consider the different scenarios in the vignettes, which enriched the analysis with different and carefully considered perspectives. One limitation that must be pointed out, however, is that age span of the female interviewees was rather narrow, as the oldest female interviewee was only 46 years of age, it could have provided further insights if we had been able to include an older female, since older people have higher tendency of having complex multimorbidity [1]. Unfortunately, we were not able to recruit an older female in the time period of the study.

Thirdly, it is a limitation that most of the interviews were performed by the second author and the analysis was made primarily by the first author, where some thoughts and ideas may have been lost in the process. Conversely, the two authors working together on the transcriptions, analysis and presentation of the results led to thorough discussion on the themes and topics that emerged, which may have led to further insight and enhanced trustworthiness.

This study is a step towards expanding our knowledge on patient perspectives on data sharing specifically regarding using AI in general practice, which is currently a very limited field. The perspectives shown in this pilot study can be used as focal points for future research on implementing and using AI in general practice and the mentioned concerns need to be taken seriously if patients are to trust the use of AI in general practice. Future research could advantageously carry out focus group interviews mixed with patients and GPs, so both patients and GPs could get a deeper understanding of each other’s viewpoints and in that way cooperate in finding ways of implementing and using AI in general practice that works for both parties. Furthermore, a larger study of GPs and patients in general practice could tease out further concerns identified in this study.

## Conclusion

The interviewees agreed that they would share their health data with their GP and that a research project on GPs using AI in general practice was a sufficient cause for which they would share their health data. All the interviewees found that AI could be an efficient and beneficial support tool for the GP in general practice. It was pointed out that patients cannot have a trustful relationship with a machine, which was mostly how the interviewees viewed AI, and therefore the interviewees insisted that the GP should continue playing a primary role in the decision making of the general practice workflow. It can therefore be argued that if AI is implemented in a way that preserves the patient-GP relationship and is used primarily as a support tool for the GP, patients may trust AI in general practice in the future.

## Electronic Supplementary Material

Below is the link to the electronic supplementary material

Supplementary Material 1

Supplementary Material 2

## Data Availability

The datasets generated and analyzed during the study are not publicly available due to assurance of data anonymization. Available upon reasonable request to Janus Laust Thomsen at jlt@dcm.aau.dk

## List of Abbreviations

AI: Artificial intelligence
AAU: Aalborg University
GP: General practitioner
