# Supplementary material for: Patient perspectives on data sharing regarding implementing and using artificial intelligence in general practice – a qualitative study
Source: BMC Health Serv Res. 2023 Apr 4;23:335. doi: 10.1186/s12913-023-09324-8 (PMC10071604; doi:10.1186/s12913-023-09324-8)
Supplement: Supplementary file 1 — Supplementary Material 1 [file 12913_2023_9324_MOESM1_ESM.docx]

**Vignettes**

The patient referred to in the vignettes is fictional.

1.
At a consultation at her GP, Anette Jensen is told about a new project where AI is being developed and tried out in the general practice. The IT-solution that builds on AI is based on patients’ health data. The intention with AI in the general practice is that it should be a helping hand in the GPs work.

2.
In the same relation Annette Jensen gets asked if her health data can be shared to develop and test AI in general practice. Annette gets told, that the wanted health data includes everything in her health journal (for example her age (58 years), profession (early retiree), that she suffers from chronical backpain, has diabetes 2 and that she earlier has had a depression), her test results (for example blood test answers and results on depression tests) and her calendar schedule that includes consultation timepoints, reservation times for blood tests etc. The GP assures Annette that her health data will be made unrecognizable in a degree that her data could not be tracked back to her or used in any other link than the concrete project. Furthermore, all the health data will be deleted after the end of the project. Annette is also reassured that only a limited number of scientists will have access to her data after the data have been made unrecognizable. Lastly an agreement of consent that needs to be signed is presented to Annette if she decides to share her health data. In the agreement of consent Annette’s rights is described, including the possibility to regret the sharing of her health data.

3.
Annette Jensen is at a consultation with her GP regarding pain in her lower body. The GP talks to Annette about the course of the pain and then examines Annette. When the GP is done, he says that he will have to do some calculations on the computer that uses AI. Annette can also see the screen while the GP lets the computer work. A lot of numbers and words popup on the screen and the GP studies them. After a while the GP looks at Annette and says: “*Well Annette. It looks like I suspected. According to my examine and the calculations performed by AI it looks like you have…”.*
