# Supplementary material for: Patient perspectives on data sharing regarding implementing and using artificial intelligence in general practice – a qualitative study
Source: BMC Health Serv Res. 2023 Apr 4;23:335. doi: 10.1186/s12913-023-09324-8 (PMC10071604; doi:10.1186/s12913-023-09324-8)
Supplement: Supplementary file 2 — Supplementary Material 2 [file 12913_2023_9324_MOESM2_ESM.docx]

# Understanding of data and artificial intelligence (AI)

Case 1
The patient referred to in the vignettes is fictional.

At a consultation at her GP, Anette Jensen is told about a new project where AI is being developed and tried out in the general practice. The IT-solution that builds on AI is based on patients’ health data. The intention with AI in the general practice is that it should be a helping hand in the GPs work.

| **Theme** | **Questions** |
| --- | --- |
| Understanding of the case | - Do you have any questions regarding the case? - Are there any elements of the case that you do not understand? - Are there any words in the case that you do not understand? |
| How/what is your understanding of data? | - What do you understand regarding health data? What is health data? Please give examples. - Which health data does the general practice keep? Please give examples. - What can data be used for? Please give examples. |
| How/what is your understanding of AI? | - How do you define AI? What is AI? Please give examples. - What can AI be used for? Please give examples. - How do you imagine AI being used in general practice? Please give examples. - How do you imagine AI helping the GP? Please give examples. - Which health data do you believe is required to develop AI in general practice? Please give examples. |

# The use of data for developing AI in general practice

Case 2

In the same relation Annette Jensen gets asked if her health data can be shared to develop and test AI in general practice. Annette gets told, that the wanted health data includes everything in her health journal (for example her age (58 years), profession (early retiree), that she suffers from chronical backpain, has diabetes 2 and that she earlier has had a depression), her test results (for example blood test answers and results on depression tests) and her calendar schedule that includes consultation timepoints, reservation times for blood tests etc. The GP assures Annette that her health data will be made unrecognizable in a degree that her data could not be tracked back to her or used in any other link than the concrete project. Furthermore, all the health data will be deleted after the end of the project. Annette is also reassured that only a limited number of scientists will have access to her data after the data have been made unrecognizable. Lastly an agreement of consent that needs to be signed is presented to Annette if she decides to share her health data. In the agreement of consent Annette’s rights is described, including the possibility to regret the sharing of her health data.

| **Theme** | **Questions** |
| --- | --- |
| Understanding of the case | - Do you have any questions regarding the case? - Are there any elements of the case that you do not understand? - Are there any words in the case that you do not understand? |
| When should data be available? | - The GP asks Annette if she wants to share her health data. - What do you think about that? Is it okay to ask for health data in this way? Why? Elaborate. - Does it have any influence that it is the GP asking for her health data? Would it have the same influence if somebody else asked, for example a secretary or a researcher? - Have you ever been in a situation like the one Annette is in, where you have been asked to share your health data or personal data? - If yes, will you elaborate? What happened? - If no, what would you think about being in a situation like that? How would you react? - Is there a difference in sharing health data and personal data? - Annette is asked to share her data for a project regarding developing AI in general practice. - In general: Do you think a projects purpose influences the decision to share data? Elaborate. - Is there any projects that are more ”legal” or ”valid” than others in relation to sharing health data? - What do you think about the project that Annette is asked to share her data for? Is it a project that is worth sharing your data for? - The GP asks Annette for permission to use her health data – which is a legal requirement – but are there projects that are so serious that is would be okay to use health data without asking the patient? For example, to make a cure for a disease, research projects. - Do you think there should always be asked permission before using a patient’s health data? No matter the purpose of the project? - What would you say if you were asked to share your health data to this project? – Developing AI in general practice. |
| What kind of data should be available? Sensitive data. | - The GP asks to use all the data in Annette’s’ journal (age, profession, current and former medical history, test results). - What do you think about the fact that the GP asks for all the data in the journal? - Is there any of the health data that are more sensitive or secret than others? Why? Why not? - What is it that makes Annette’s health data sensitive? - Does the degree of sensitivity influence the willingness to share health data? |
| How safe should data be? (Data security) | - The GP assures a certain degree of security and protection regarding Annette’s health data (recognition, tracing, consent). - Do you think the protection is adequate? Why? Why not? - Is there missing information regarding the protection of Annette’s health data? Which? Why? - How does the assurance of protection influence Annette’s decision to share her health data? According to you. - What about the more sensitive data, how does the assurance of protection influence the willingness to share these? - Annette is assured, that her health data will not be shared with anyone other than the involved parties (the researcher, the GP) - What do you think about that? Elaborate. - Should there be any limits regarding who data gets shared with? - Is there anyone that must not get access to health data? Examples. |
| Follow-up on theme | - Do you have any perspectives that you would like to talk more about or elaborate on? |

# The use of AI in general practice

Case 3

Annette Jensen is at a consultation with her GP regarding pain in her lower body. The GP talks to Annette about the course of the pain and then examines Annette. When the GP is done, he says that he will have to do some calculations on the computer that uses AI. Annette can also see the screen while the GP lets the computer work. A lot of numbers and words popup on the screen and the GP studies them. After a while the GP looks at Annette and says: “*Well Annette. It looks like I suspected. According to my examine and the calculations performed by AI it looks like you have…”.*

| **Theme** | **Questions** |
| --- | --- |
| Understanding of case | - Do you have any questions regarding the case? - Are there any elements of the case that you do not understand? - Are there any words in the case that you do not understand? |
| Trust regarding the use of AI in general practice. | - What do you think about the case? - The GP uses AI as a support tool after the examination of Annette. - How do you feel about AI supporting the GPs work? - How do you think AI will influence the GPs workflow? Would it change anything? Would it have a positive or negative effect on the GPs work? - Would AI supporting the GPs work affect your trust in the GPs work? - Would you trust the treatment more or less if AI was in the picture? - In the case it seems like Annette is ill. - Do you think the GP should trust AI every time, in every scenario? - When is it okay for the GP to use AI in relation to detect diseases that would not otherwise have been detected? - If AI predicted that Annette gets very ill within a short amount of time, should the GP react? - What about future illnesses? (15-20 years in the future)? - Let us imagine that AI detects a serious disease, that does not correlate with Annette’s actual reason for seeking out the GP. - Should the GP react? How? Why? - When is it okay to interfere with Annette’s life without prior inquiry? |
| Conclusion of interview | - I do not have any more questions. Do you have something to add before we end the interview? |
